# The molecular significance of methylated *BRCA1* promoter in white blood cells of cancer-free females

**DOI:** 10.1186/1471-2407-14-830

**Published:** 2014-11-17

**Authors:** Nisreen Al-Moghrabi, Asmaa Nofel, Nujoud Al-Yousef, Safia Madkhali, Suad M Bin Amer, Ayodele Alaiya, Zakia Shinwari, Taher Al-Tweigeri, Bedri Karakas, Asma Tulbah, Abdelilah Aboussekhra

**Affiliations:** Department of Molecular Oncology, King Faisal Specialist Hospital and Research Center, PO BOX 3354, 11211 Riyadh, Kingdom of Saudi Arabia

**Keywords:** Breast cancer, *BRCA1*, Methylation, White blood cells, Gene expression

## Abstract

**Background:**

*BRCA1* promoter methylation has been detected in DNA from peripheral blood cells of both breast cancer patients and cancer-free females. However, the pathological significance of this epigenetic change in white blood cells (WBC) remains an open question. In this study, we hypothesized that if constitutional *BRCA1* methylation reflects an elevated risk for developing breast cancer (BC), WBC that harbor methylated *BRCA1* in both cancer-free females and BC patients should exhibit similar molecular changes.

**Methods:**

*BRCA1* promoter methylation was examined by methylation-specific PCR in WBC from 155 breast cancer patients and 143 cancer-free females. The Human Breast Cancer EpiTect Methyl II Signature PCR Array and The Human Breast Cancer RT^2^ Profiler™ PCR Array were used to study the methylation status and the expression profile of several breast cancer-related genes, respectively. In addition, we used label-free MS-based technique to study protein expression in plasma.

**Results:**

We have shown that 14.2% of BC patients and 9.1% of cancer-free females (carriers) harbored methylated *BRCA1* promoter in their WBC. Interestingly, 66.7% of patients harbored methylated *BRCA1* promoter in both WBC and tumors. Importantly, we have shown the presence of epigenetic changes in 9 other BC-related genes in WBC of both patients and carriers. Additionally, *BRCA1* and 15 other important cancer –related genes were found to be differentially expressed in WBC from patients and carriers as compared to controls. Furthermore, we have shown that the carriers exhibited a unique plasma protein pattern different from those of BC patients and controls, with 10 proteins similarly differentially expressed in patients and carriers as compared to controls.

**Conclusions:**

The present results suggest the presence of a strong link between aberrant methylation of the *BRCA1* promoter in WBC and breast cancer –related molecular changes, which indicate the potential predisposition of the carriers for developing breast cancer. This informs the potential use of the aberrant methylation of *BRCA1* promoter in WBC as a powerful non-invasive molecular marker for detecting predisposed individuals at a very early age.

## Background

Epigenetic is the inheritance of information on the basis of gene expression rather than direct changes to sequence composition [1]. Errors in epigenetic regulation, which result in aberrant transcriptional silencing of a normally active gene or reactivation of a normally silent gene, are termed *epimutations*[2]. In human cancers, this heritable yet non-genetic modification is a powerful mechanism responsible for the inhibition of different types of genes, including tumor suppressor genes [3]. Epimutation that is found in all tissues of the body could be either germline, with evidence of inheritance, or constitutional, no evidence of inheritance. While it is still controversial whether germline epimutations occur in humans [4–6], constitutional epimutation is increasingly being considered as a mechanism for cancer predisposition.

Breast Cancer Associated gene1, *BRCA1*, was identified in 1994 as the first gene associated with familial breast cancer predisposition [7]. Since then, germline mutations of *BRCA1* have been found to be responsible for the hereditary type of breast cancer, which accounts for about 5-10% of all breast cancers. Individuals carrying germline *BRCA1* mutations are more likely to develop aggressive breast tumors at an early age (<50) [8]. These tumors are characterized by aneuploidy, high grade, poor histologic differentiation, and the majorities are of the triple-negative subtype, which is negative for estrogen receptor (ER), progesterone receptor (PR) and HER2 expression [8]. Gene silencing by epigenetic mechanisms is an alternative mechanism for *BRCA1* inactivation during sporadic carcinogenesis [9]. Results from various studies revealed that 9-44% of sporadic breast cancer samples harbor hypermethylated *BRCA1* promoter [9, 10]. The pathological features of these tumors are similar to those with inherited mutated *BRCA1*. Indeed, both occur at an early age and present poor histological differentiation, aneuploidy, ER and PR negativity, as well as similarities in their global gene expression profiles [11, 12].

Lately, *BRCA1* promoter methylation has been detected in DNA from both white blood cells and tumor tissues in 3 out of 7 breast cancer patients from breast-ovarian cancer families [13]. This suggested that *BRCA1* promoter methylation occurring in normal tissue of the body is associated with the development of *BRCA1*-like breast cancer [13]. Similarly, we have recently reported *BRCA1* promoter hypermethylation in WBC genomic DNA of 2 out of 7 (28.5%) breast cancer patients, whose tumors showed *BRCA1*-like characteristics [14]. Furthermore, we have shown *BRCA1* methylation in WBC of 8 out of 73 (10.9%) cancer-free women. The presence of methylated *BRCA1* promoter in healthy females may reveal predisposition of these individuals to develop breast cancer. Indeed, the functional equivalence between the effect and significance of the epigenetic silencing of *BRCA1* and the inheritance of *BRCA1* mutations [15–17], has supported the notion that *BRCA1* promoter methylation may serve as a first hit, much like an inherited germline mutation.

Although several studies have indicated the association between the presence of methylated *BRCA1* promoter in WBC and the risk of developing breast cancer [18–20], the pathological significance of methylated *BRCA1* promoter in WBC of cancer-free women remains still unclear. In the present study, we provide clear evidences that females with methylated *BRCA*1 in their WBC have, epigenetic changes, modulated gene expression profile and changes in protein expression in plasma similar to that seen in *BRCA1*-methylated breast cancer patients, advocating the possible involvement of *BRCA1* constitutional epimutation as an alternative breast cancer predisposition mechanism.

## Methods

### Study samples

Breast cancer: 10 ml fresh blood samples were collected from 155 breast cancer female patients coming to the oncology department in King Faisal Specialist Hospital and Research Centre in Riyadh, Saudi Arabia. Paraffin embedded breast cancer tissues were obtained from the Department of Pathology. The age of the patients at diagnosis ranged from 23 to 73 years. Clinicopathological data (age, histological grade and ER and PR status) were provided by the Department of Pathology. Control samples: 10 ml fresh blood samples were collected from 143 healthy cancer-free female volunteers with ages ranged from 15 to 47 years. All patients and controls gave written informed consent to participate in the study. The study was approved by the Human Research Ethics Committee of King Faisal Specialist Hospital and Research Centre.

### Isolation of DNA and RNA from WBC

Fresh blood was collected in 2 EDTA blood collection tubes. The tubes were centrifuged immediately at 3000 rpm for 10 min at 4°C. The WBC layers were carefully collected and transferred into two 2 ml Eppendorf tubes, one containing 900 mls RBC Lysis solution for subsequent DNA extraction using the Gentra Puregene Blood Kit, and the other tube contained 1.2 ml RNALater solution for subsequent RNA extraction using RiboPure Blood Kit (Ambion).

### Isolation of DNA from paraffin embedded tissues

Genomic DNA was isolated from two to three 10 μM thick paraffin sections using Puregene kit (Gentra).

### Methylation specific PCR

DNA methylation was assessed by methylation-specific PCR of sodium bisulfate treated DNA. 1 μg of genomic DNA was treated with sodium bisulfite and purified using EpiTect Bisulfite Kit (Qiagen) following the manufacturer’s recommendations. Modified DNA was amplified with published PCR primers for *BRCA1 and MGMT*[9, 21] that distinguish methylated and unmethylated DNA. PCR products were electrophoresed on 2% agarose gels and stained with Ethidium bromide. SssI methylase treated and untreated bisulfite modified DNA was used as positive and negative controls, respectively. All PCR reactions were done in replicates.

### Methylation PCR array

The Human Breast Cancer EpiTect Methyl II Signature PCR Array (Qiagen) was used to study the methylation status of 24 different breast cancer-related genes. 1 μg of genomic DNA isolated from WBC was used in the array following the manufacturer protocol. Data analysis was done using integrated Excel-based templates provided by the manufacturer, which provide gene methylation status as percentage unmethylated (UM) and percentage methylated (M) fraction of input DNA. M" represents the fraction of input genomic DNA containing two or more methylated CpG sites in the targeted region of a gene. 2.5-fold or greater change relative to controls was determined to be the threshold cut-off point for what is considered a change in gene methylation.

### RT^2^ Profiler™ PCR array

The Human Breast Cancer RT^2^ Profiler™ PCR Array was used to profile the expression of 84 different breast cancer-related genes. 1 μg of total RNA isolated from WBC was revers-transcribed into cDNA using the RT^2^ first strand kit (SABiosciences) following the manufacturer’s instructions, which was then used in the Array. The Array was set following the manufacturer’s instructions and was performed according to the manufacturer’s protocol. Data analysis was done using online software PCR Array Data Analysis Web Portal provided by the manufacturer. Gene expression levels were normalized against five housekeeping genes included in the Array. Fold changes in gene expression were calculated using the 2-∆∆Ct method by the software. 1.5-fold or greater change was determined to be the threshold cut-off point for what is considered a change in gene expression.

### In solution-digestion and protein identification by mass spectrometry: LC-MS^E^ analysis

Plasma samples from 4 breast cancer patients, 4 carriers and 4 controls were handled and prepared similarly. Equal amount of proteins was taken from each sample to generate a pool of patients as one group, a pool of carriers, and a third pool of controls. For each analysis sample group, 200 μg complex protein mixtures was subjected to in-solution digestion for mass spectrometry analysis as previously described [22, 23]. We have used the 1-Dimensional Nano Acquity liquid chromatography coupled with tandem mass spectrometry on Synapt G2 (Waters, Manchester, UK) to generate expression proteomics data based on quantitative protein changes between the three sample groups. The ESI- MS analysis and instrument settings were optimized on the tune page as previously described [22, 23]. All samples were analyzed in triplicate runs and data were acquired using the Mass Lynx programs (version. 4.1, SCN833, Waters, Manchester, UK) operated in resolution and positive polarity modes. Protein Lynx Global Server (PLGS) 2.5 (Waters, Manchester, UK) was used for all automated data processing and database searching. The generated peptide masses were searched against Uniprot Human protein sequence database using the PLGS 2.5 for protein identification (Waters, UK). TransOmics Informatics (Waters Corporation, UK) was used to process and search the data. The principle of the search algorithm is described [24]. The following criteria were used for the search 1 missed cleavage, Max protein mass 1000 kDa, Trypsin, Carbamidomethyl C fixed and Oxidation M variable modifications. Normalized label-free quantification was achieved using Progenesis QI software, (Nonlinear Dynamics (Newcastle, UK). The data was filtered to show only statistically significantly regulated proteins (ANOVA), (p ≤0.001) and a fold change >1.5. The data set was subjected to unsupervised PCA analysis.

### Statistical analysis

The Chi-square and Fisher’s exact tests were performed to determine the statistical significance for the correlation between *BRCA1* promoter methylation and age, and *BRCA1* promoter methylation and cancer family history. T-test was performed to determine the statistical significance between the different groups for methylation (Carriers vs controls, Patients vs controls and Carriers vs patients). ANOVA test with multiple comparisons using SAS version 9.3(SAS Institute, Cary, NC, USA) was performed for confirmation. T-test was performed to determine the statistical significance between the carriers and the patients groups for gene methylation and expression levels. All observed differences were considered to be significant when associated with a P value <0.05.

## Results

### Identification of breast cancer patients and cancer-free females harboring methylated *BRCA1*promoter in their WBC

**Figure 1:**
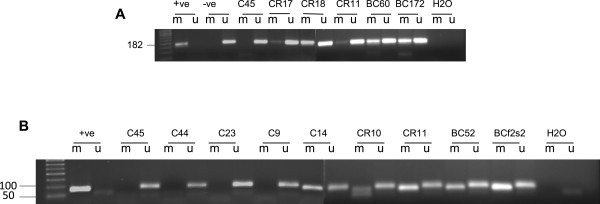
**MSP analysis assay, (A) MSP analysis of the** ***BRCA1*** **promoter region, (B) MSP analysis of the** ***MGMT*** **promoter region in WBC DNA.** *Sss*I methylase-treated and -untreated bisulfite-modified DNA was used as positive (+ve) and negative (-ve) controls, respectively. BC; breast cancer, CR; carriers and C; controls; M, methylated product; U; unmethylated product.

In order to identify breast cancer patients and cancer-free females harboring methylated *BRCA1* promoter in their WBC, we screened 155 patients and 143 cancer-free females using the methylation-specific PCR (MSP) assay. We identified 22 (14.2%) breast cancer patients harboring methylated *BRCA1* promoter (Figure 1A). Interestingly, 20 out of 22 were <50 years (90.9%), and only 2 were >50 years (9.1%) (Table 1). This indicates a strong association between the presence of methylated *BRCA1* promoter in WBC and early onset of breast cancer (p = 0.032). In addition, we identified 13 cancer-free healthy females (9.1%) harboring methylated *BRCA1* promoter in their WBC (Figure 1A). Importantly, 11 out the 13 were <40 years (84.6%), and only 2 were >40 years (15.4%) (Table 2). Additionally, we have found a significant association between the incidence of cancer in the family of those subjects and the presence of *BRCA1* promoter methylation in their WBC (10/13) 77% (p = 0.036). 7 of those families 7/10 (70%) have breast/ and or ovarian cancer history. For simplification, we termed cancer-free females harboring methylated *BRCA1* as “Carriers”.

**Table 1:** **Clinical characterizations of** ***BRCA1*** **methylated breast cancer positive cases**

Breast Cancer samples (BC)	Age	***BRCA*** 1 status in tumor tissue	ER	PR	HER-2	Breast cancer tumor type	Histological grade
13	67	+	+ve	+ve	-ve	ILC	G1
138	40	+	+ve	+ve	-ve	IDC	G2
172	41	+	+ve	-ve	-ve	IDC	G3
154	46	+	+ve	+ve	-ve	IDC	G2
176	49	+	-ve	-ve	-ve	IDC	G3
116	41	+	-ve	-ve	-ve	metaplastic	
181	42	-	+ve	+ve	-ve	IDC	G2
171	37	ND	-ve	-ve	-ve	IDC	G2
52	69	-	+ve	+ve	-ve	ILC	G1
81	27	-	+ve	-ve	+ve	IDC	G2
130	39	+	-ve	-ve	-ve	IDC	G3
60	40	+	-ve	-ve	-ve	IDC	G2
183	36	+	-ve	-ve	-ve	IDC	G2
111	44	ND	+ve	+ve	-ve	IDC	G2
161	48	ND	+ve	+ve	-ve	IDC	G2
187	33	ND	-ve	-ve	-ve	IDC	G3
54	40	-	+ve	+ve	-ve	ILC	
58	39	+	+ve	+ve	-ve	IDC	G2
86	23					Fibro adenoma benign	
S3	40	-	+ve	+ve	-ve	ILC	
S2	43		ND	ND	ND	ND	
F2S2	39		ND	ND	ND	ND	

IDC; Invasive ductal carcinoma.

ILC; Invasive lobular carcinoma.

ND; No data.

**Table 2:** **Cancer family history of the carriers**

Carriers Samples (CR)	Age	Affected family members	Type of cancer in affected relative	BRCA1 methylation in affected family member
CR7	25	Cousin	Breast cancer	ND
CR9	48	Grandmother, two aunts and cousin	Breast cancer	
CR18	41	Aunt (mother side)	Ovarian cancer	ND
CR21	17	Mother	Breast cancer	Yes
CR25	27	Aunt	Breast cancer	ND
CRF2D3	15 (Twin)	Mother and two aunts (mother side)	Breast cancer	One aunt
CR39	22	Great grandmother (mother side)	Breast cancer	ND
		Mother	Ovarian cancer	ND
CR5	28	ND	Cancer (other than breast and ovarian)	ND
CR10	27	No cancer in the family		
CR11	26	ND	Cancer (other than breast and ovarian)	ND
CR17	26	No cancer in the family		
CR35	22	ND	Cancer (other than breast and ovarian)	ND
CR59	27	NO cancer in the family		

ND; No data.

### Strong correlation between *BRCA1*methylation in WBC and matched breast tumors

Next, we sought to study the correlation between *BRCA1* promoter methylation in WBC and their paired tumors. To this end, the methylation status of the *BRCA1* promoter was studied, by the MSP assay, in genomic DNA isolated from matched tumor samples obtained from the above *BRCA1* methylated positive breast cancer cases. Out of the 22 *BRCA1* methylated positive cases, only 19 archived tumor tissues were available for *BRCA1* methylation analysis. The *BRCA1* promoter was hypermethylated in 10 samples, unmethylated in 5 samples and no results could be determined for 4 samples (Table 1). This indicates that 66.7% (10/15) of patients harbored methylated *BRCA1* promoter in both WBC and tumors, which reveals strong correlation between the presences of methylated *BRCA1* promoter in WBC and matched tumor tissues. Out of these 10 cases 5 had tumors of the triple negative type (50%).

### WBC with methylated *BRCA1*show similar epigenetic changes in both breast cancer cases and carriers

**Figure 2:**
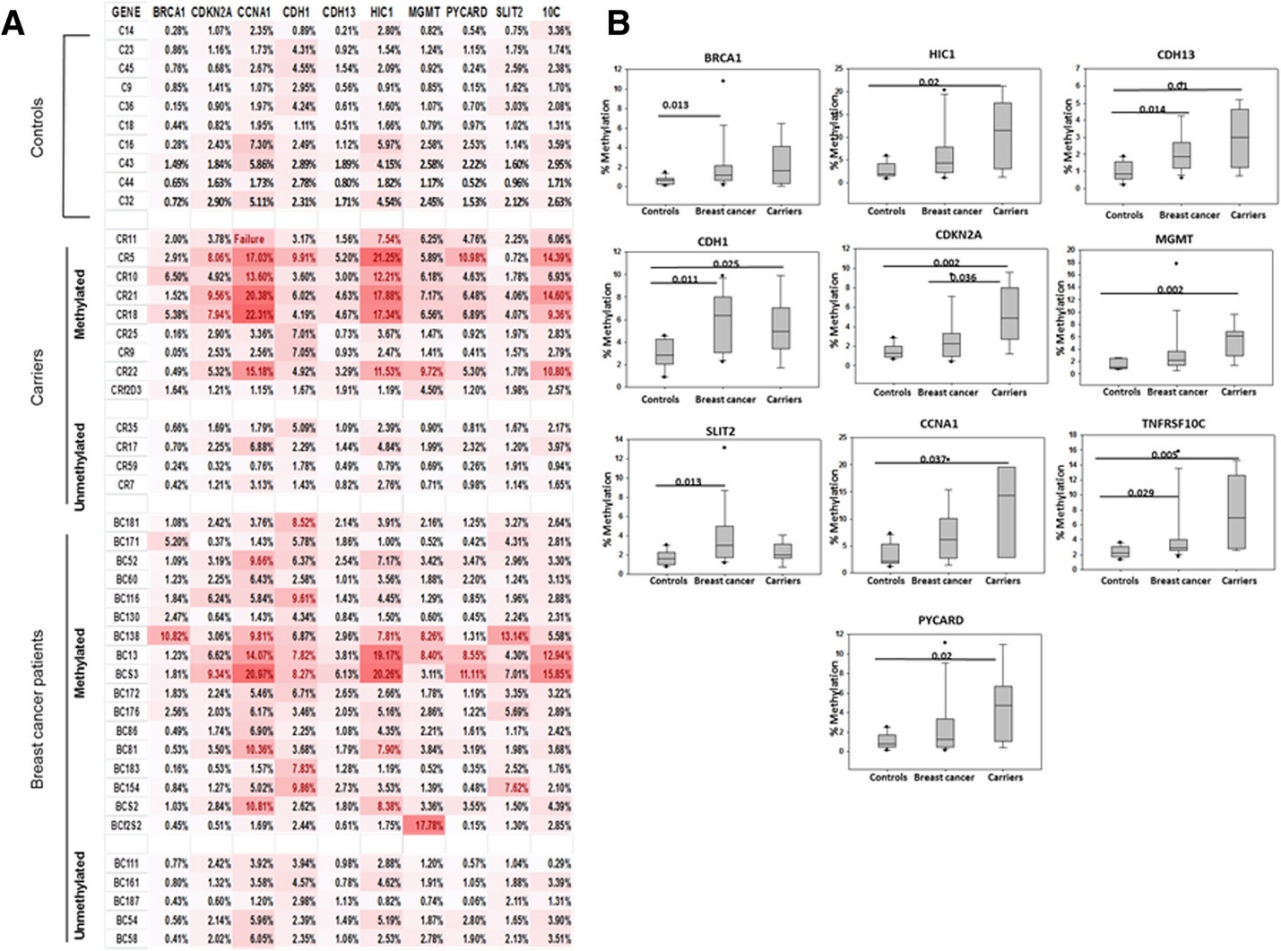
**WBC with methylated** ***BRCA1*** **show similar epigenetic changes in both breast cancer cases and carriers (A) a heat map comparing the methylation status of a panel of 10 cancer-related genes in WBC from controls C, breast cancer BC, and carriers CR as determined using Human Breast Cancer EpiTect Methyl II Signature PCR Array.** The red color represents values of +2.5 fold relative to controls **(B)** Comparison between quantitative analysis of methylation for the candidate genes in the studied cohort; Controls (n = 10), Breast cancer (n = 17) and Carriers (n = 9). Significant correlation; Chi square. Numbers represent *p* values. Error bars represent the mean ± SD.

Since *BRCA1* has been found to be methylated in WBC genomic DNA, we hypothesized that other breast cancer related genes may also be epigenetically affected. Hence, we made use of the Human Breast Cancer EpiTect Methyl II Signature PCR Array, which profiles the promoter methylation status of 24 genes, whose hypermethylation is known to play a role in breast carcinogenesis. WBC from 17 cases, 13 carriers and 10 controls were used in these experiments. On the basis of a cut-off value of +2.5 fold relative to controls, 9 different breast cancer-related genes, other than *BRCA1*, were highly methylated in the breast cancer cases compared to controls (Figure 2), *HIC1* (p = 0.093), *CDH13* (p = 0.014), *CDH1* (p = 0.011), *CDKN2A* (p = 0.167), *MGMT* (p = 0.067), *SLIT2* (p = 0.013), *CCNA1* (p = 0.075), *TNFRSF10C* (p = 0.029) and *PYCARD* (p = 0.269). Interestingly, the same genes, except *SLIT2*, were also highly methylated in the carriers compared to controls (Figure 2), *HIC1* (p = 0.02), *CDH13* (p = 0.01), *CDH1* (p = 0.025), *CDKNA2* (p = 0.002), *MGMT* (p = 0.002), *CCNA1* (p = 0.037), *TNFRSF10C* (p = 0.005) and *PYCARD* (p = 0.02). This indicates that WBC harboring *BRCA1* promoter methylation exhibit similar epigenetic changes in both breast cancer patients and cancer-free females. On the other hand, WBC from 5 breast cancer cases and 4 carriers did not show any changes in methylation levels for the 9 genes as compared to controls (Figure 2A).

In order to validate the signature PCR Array data, the methylation status of *MGMT* was analyzed by MSP in five control samples, two breast cancer cases, and two carriers. The *MGMT* methylated band was detected in WBC of all breast cancer cases and carrier samples. However, only one control sample showed the presence of a methylated *MGMT* band (Figure 1B).

### Similar gene expression pattern in WBC with methylated *BRCA1*from breast cancer cases and carriers

**Figure 3:**
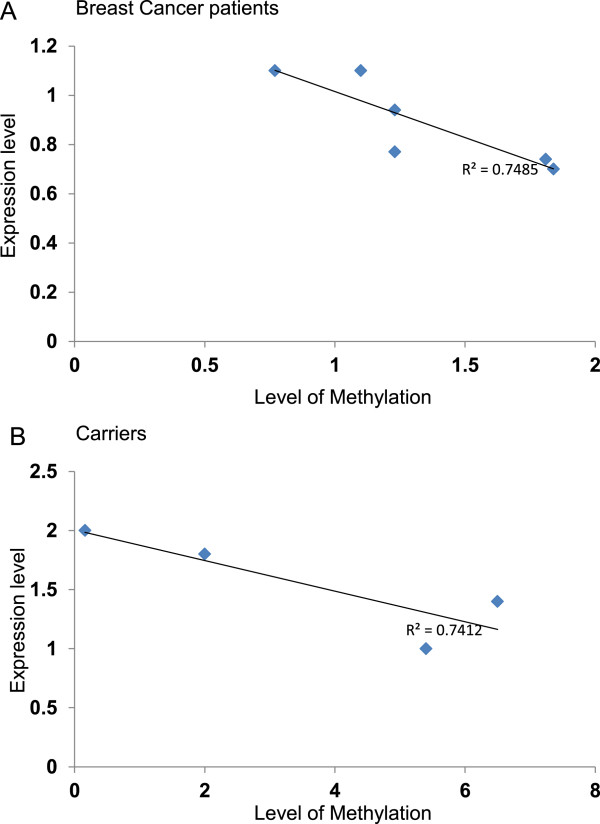
**Correlations of the mRNA and methylation levels of** ***BRCA1*** **.** Correlation between the methylation levels of *BRCA1* in WBC determined by Human Breast Cancer EpiTect Methyl II Signature PCR Array and the mRNA levels measured by Human Breast Cancer RT^2^ Profiler™ PCR Array **(A)** breast cancer group **(B)** carriers group, R^2^; correlation coefficient.

**Figure 4:**
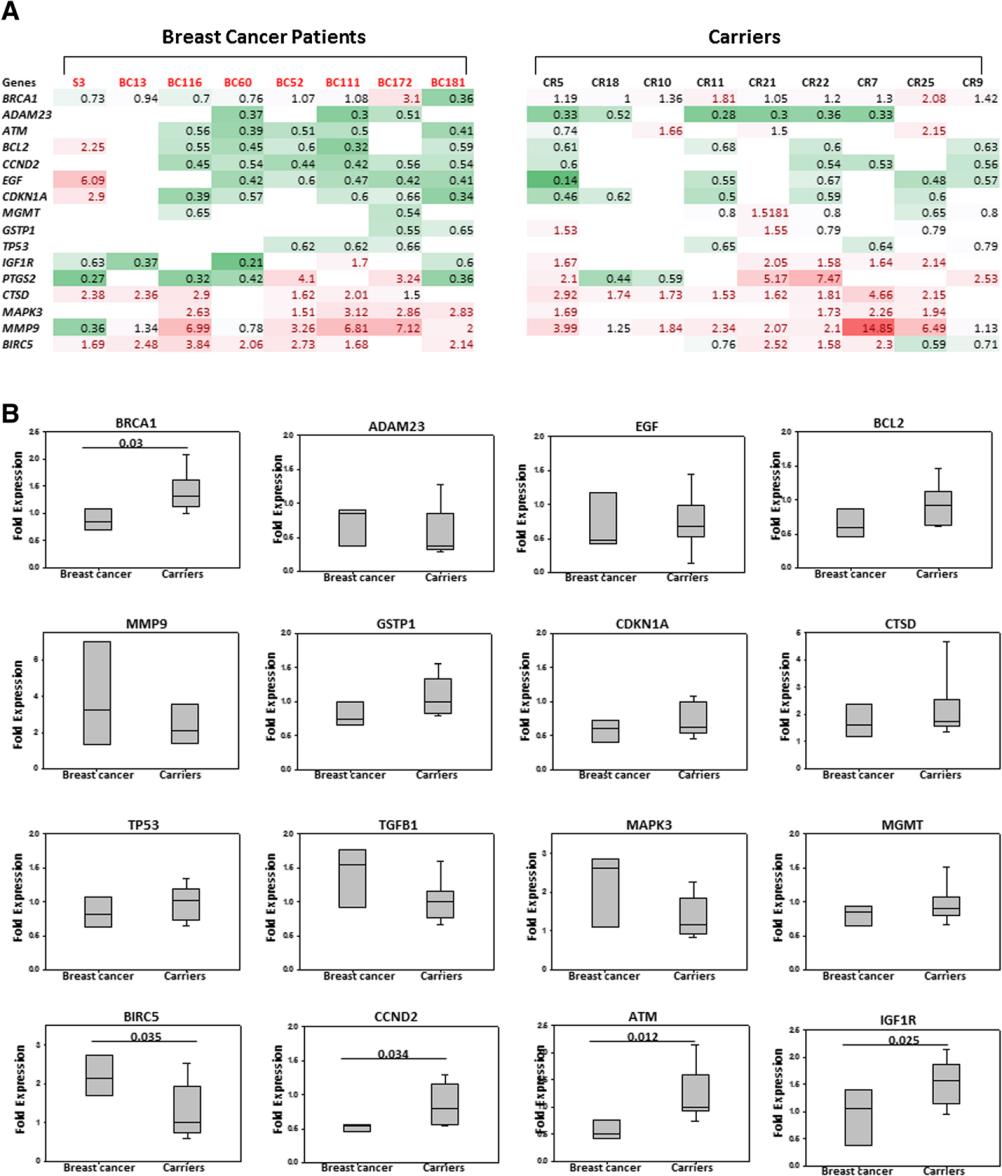
**Similar gene expression pattern in WBC with methylated** ***BRCA1*** **from breast cancer cases and carriers (A) a heat map comparing the differential expression of16 cancer-related genes in WBC RNA from breast cancer patients and carriers as compared to controls determined by using Human Breast Cancer RT** ^**2**^ **Profiler™ PCR Array. (B)** Comparison between quantitative analysis of expression level for the candidate genes in the studied cohort; Breast cancer (n = 8), Carriers (n = 9) against controls (n = 5). Significant correlation; Chi square. Numbers represent *p* values. Error bars represent the mean ± SD.

Next, we sought to assess the expression of cancer-related genes, including *BRCA1*, at the mRNA level, in the WBC that harbor methylated *BRCA1* promoter. To this end, we made use of the Human Breast Cancer RT^2^ Profiler™ PCR Array that profiles the expression of 84 key genes, commonly involved in breast carcinogenesis, using RNA isolated from WBC of 8 breast cancer cases, 9 carriers and 5 controls. Out of 84 genes present in the array, 17 were not detected in the WBC. Importantly, we have found a strong correlation between the expression level of *BRCA1* in breast cancer cases and its methylation levels detected by the Methyl II Signature PCR Array (Figure 3A). However, only 4 samples from the carriers group showed such correlation, CR10, CR11, CR18 and CR25 (Figure 3B). On the basis of the cut-off value ±1.5 fold relative to controls, 16 genes, including *BRCA1*, were differentially expressed in breast cancer cases and carriers as compared to controls (Figure 4A). Interestingly, no significant differences were detected between the breast cancer cases and the carriers for the expression of 11 different genes:, *ADAM23* (p = 0.343), *BCL2* (0.213), *EGF* (p = 0.90), *CDKN1A* (p = 0.567), *CTSD* (0.397), *GSTP1* (0.186), *MAPK3* (p = 0.058), *MGMT* (p = 0.165), *MMP9* (p = 0.463), *TGFB1* (p = 0.084) and *TP53* (p = 0.334) (Figure 4B). This indicates that WBC from cancer-free females with methylated *BRCA1* has abnormal gene expression profile similar to that seen in WBC from breast cancer cases. On the other hand, 5 genes were differentially expressed with significant difference between the breast cancer patients and carriers, *BRCA1* (p = 0.03), *BIRC5* (p = 0.035), *CCND2* (p0.034), *ATM* (p = 0.012) and *IGF1R* (p = 0.025) (Figure 4B).

### Similar changes in protein expression pattern in plasma from patients and carriers with WBC harboring methylated *BRCA1*

**Figure 5:**
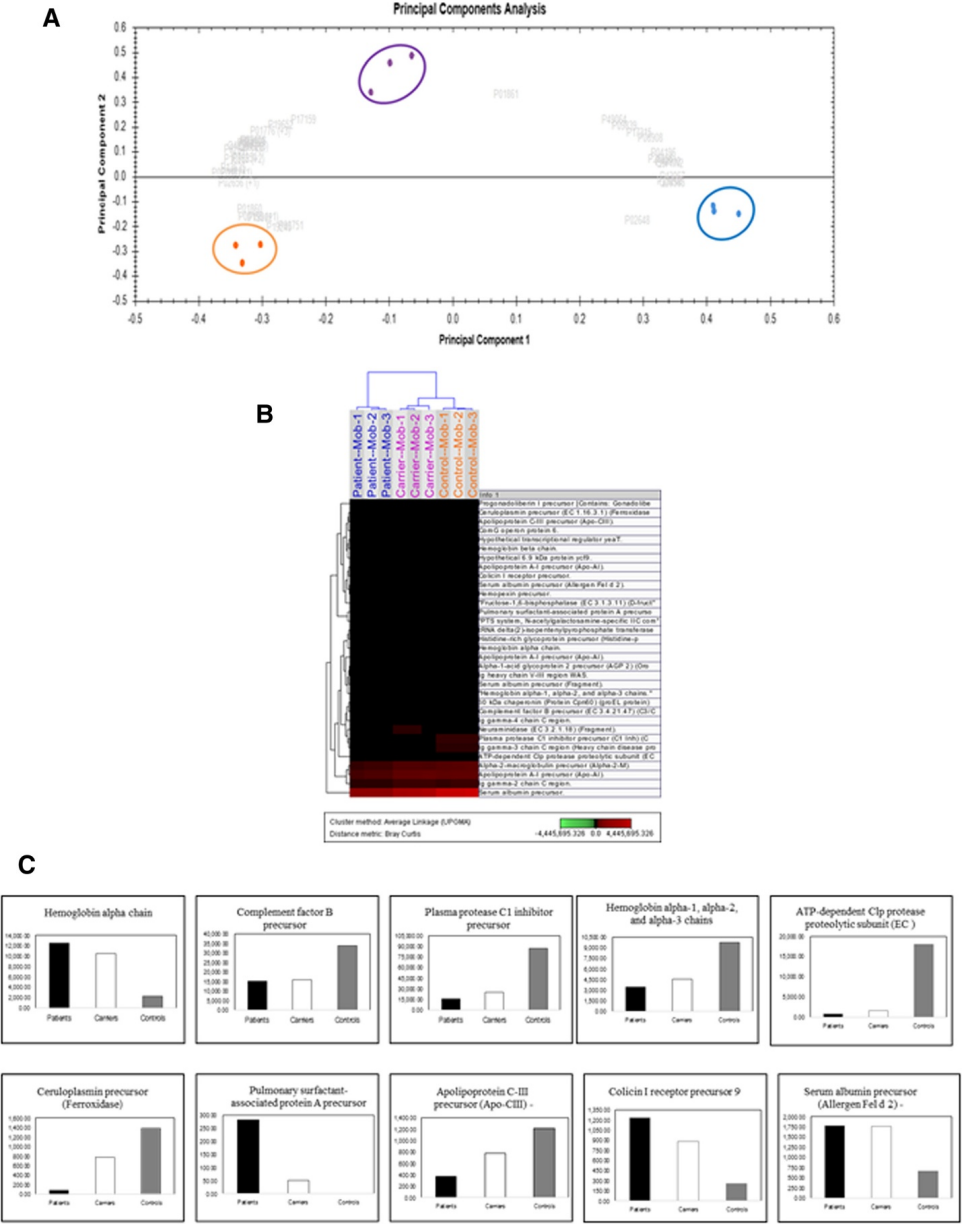
**Similar changes in protein expression pattern in plasma from patients and carriers (A) Principal Component Analysis using the 35 identified proteins with significant difference in expression between plasma samples from breast cancer patients, carrier and controls.** The accession numbers of the identified proteins are indicated in the grey color, while Blue, cancer, Purple, carrier, and Orange, control. **(B)** Hierarchical Cluster Analysis using the expression profiles of the 35 identified proteins with significant difference between plasma samples from breast cancer patients, carrier and controls. (Blue, cancer, Purple, carrier, and Orange, control). **(C)** Histograms showing relative expression (quantitation), based on intensities, of10 out of the 35 differentially expressed proteins (The expression changes/ abundance ratio were calculated based on the averaged intensities of all identified peptides corresponding to an identification of a particular protein). (The proteins were identified by LC/MS/MS/Synapt G2 and the expression data was generated using Progenesis LC-MS/QI by Nonlinear Dynamics)”. The numbers on the x axis represent Normalized Averaged Intensities.

It is anticipated that combination of different analyses platforms would give better and more reliable information to answer complex biological questions. Hence, we sought to investigate the protein plasma signature in the carriers group and compare it with those from breast cancer cases and controls. To this end, we have used the label-free MS-based technique as a tool for quantitative and comparative expression analysis. Approximately 400–450 proteins were identified during each run across all sample groups. This resulted in 91 unique protein species from all three sample groups. Only 35 of these proteins were found to be differentially expressed with significant expression changes of at least 1.5 fold and with a probability of ≥0.95-1, between patient, carrier and control sample groups. This dataset discriminated the samples into three distinct groups by unsupervised principal component analysis (PCA) and Hierarchical Cluster Analysis (Figure 5A, B). This indicates that the carriers group exhibits a unique plasma protein pattern different from those of breast cancer patients and controls. Importantly, 10 out of the 35 differentially expressed proteins showed similar expression patterns in patients and carriers as compared to controls (Figure 5C). This indicates that cancer-free females with WBC methylated *BRCA1* have abnormal plasma protein expression profile with great similarities with that seen in plasma from the breast cancer cases.

## Discussion

Up to date, the molecular and the pathological significance of the presence of methylated *BRCA1* promoter in WBC has not been investigated. The main aim of the present study was to investigate, at the molecular level, whether women with methylated *BRCA1* promoter in their WBC are indeed at an elevated risk of developing breast cancer. We have shown that the *BRCA1* promoter is methylated in WBC of 14.2% of breast cancer patients and that this methylation is significantly associated with the early onset of the disease. These results are in agreement with previous studies, which showed that *BRCA1* epimutations were significantly enriched in woman with early-onset breast cancer [20].

While there is significant correlation between *BRCA1* mutation and development of breast cancer, it is still controversial whether or not there is a correlation between the presence of methylated *BRCA1* promoter in WBC and paired tumor DNA from breast cancer patients [25]. In our study, we have shown that 10 out of 15 (66.7%) breast cancer patients, with methylated *BRCA1* in their WBC, displayed *BRCA1* methylation in paired tumor DNA. This suggests that methylated *BRCA1* in WBC may also trigger the formation of breast cancer. Indeed, it has been hypothesized that constitutional *BRCA1* methylation may constitute the “first-hit” predisposing and initiating tumorigenesis with morphologic features similar to those related to *BRCA1* germline mutations [19]. However, in patients with no methylated *BRCA*1 in paired tumour DNA, *BRCA1* promoter methylation is only specific to WBC and does not directly contribute to breast cancer of those patients [25]. Nonetheless, the fact that we have observed similar molecular changes, in the WBC from the two groups of patients, suggests a strong link between aberrant methylation of *BRCA1* promoter in WBC and breast carcinogenesis, regardless of its presence or absence in breast tissue.

To further appreciate the link between *BRCA1* promoter methylation in WBC and breast carcinogenesis we searched for *BRCA1* methylation in WBC from cancer-free women. Since, in our previous study, we have found a strong association between *BRCA1* promoter methylation and young age (≤40 years) at diagnosis [14], it was crucial to screen for methylated *BRCA1* promoter in cancer-free control cohort of young age. Interestingly, we have shown that *BRCA1*promoter is methylated in WBC of 9.1% cancer-free females. Importantly, the majority of those carriers (84%) are <40 years, which is to the contrary of the breast cancer cases where the majority of the patients are >40 (91%). This might be an indication for the potential predisposition of those individuals for developing breast cancer. Interestingly, we have found that 10/13 (77%) (p = 0.036) of the carriers have cancer family history. Importantly, 7 of those families 7/10 (70%) have breast and/ or ovarian cancer history.

In this study, we hypothesized that if *BRCA1* methylation in WBC reflects an elevated risk for developing breast cancer, WBC from the carriers should exhibit molecular changes similar, to some extent, to those described in *BRCA1*-methylted WBC from breast cancer patients. Indeed, we have shown significant epigenetic changes in 9 different breast cancer-related genes, other than *BRCA1*, in WBC from both the carriers and breast cancer patients. All of these genes are known to be involved in different aspects of breast carcinogenesis, which includes tumor suppression, *HIC1*[26], *CDH1*[27], *CDH13*[28], *CDKN2*[29], DNA repair, *MGMT*[30], apoptosis, *PYCARD*[31], *TNFRSF10C*[32], and cell cycle regulation, *CCNA1*[33]. In addition, we report the differential expression of another fifteen cancer-related genes, other than *BRCA1*, in the WBC from the carriers and breast cancer patients. This is very interesting as it has always been known that many of the “cancer-related” genes are tissue-specific and seem unlikely to pop out of WBC analyses. Nevertheless, our results demonstrate the potential use of the WBC as an important source for profiling cancer-related gene expression.

In order to validate our results, we first assessed the methylation status and the expression level of *BRCA1* in WBC from the two studied groups. As expected, the level of *BRCA1* methylation was significantly higher in WBC from breast cancer group than from the carriers. This was further confirmed by showing that the *BRCA1* mRNA was significantly lower in the breast cancer group than in the carriers. This is in concordance with the fact that constitutional methylation is mono allelic [25], hence only one allele of the *BRCA1* gene is methylated in the carriers, however, in the breast cancer patients, the two alleles are affected (according to Knudsen’s hypothesis of tumor suppressor deactivation) [34].

One of the important genes that we have shown to be differentially expressed in the WBC from the carriers and breast cancer patients is *ATM*, which is a risk factor for breast cancer [35]. This gene is down- regulated in breast tumours confirming its potential role in the development of such tumours [35, 36]. Intriguingly, we have shown a significant increase in the mRNA level of *ATM* in the WBC from the carries as compared to that in the breast cancer cases. In fact, *ATM* is also up regulated in cases of sclerosing adenosis (SA) [37], a benign proliferative disease of the breast, which is an independent risk factor for subsequent invasive ductal carcinomas [38]. This suggests that the increase in the mRNA level of *ATM* in the WBC of the carriers could be an indication for the potential predisposition of those individuals for developing breast cancer.

The insulin-like growth factor receptor (*IGF1R*) is another important gene that we have shown to be differentially expressed in WBC from the carriers and breast cancer patients. An elevation in the expression of *IGF1R* in normal breast tissue is known to be associated with an increase in the risk of subsequent breast cancer [39]. However, this gene is down-regulated in advanced human breast cancer [40]. Similarly, the expression of *IGF1R* mRNA was found to be down-regulated in peripheral blood cells and stimulated monocytes from patients with advanced stages of colorectal carcinoma (CRC) [41]. Conversely, *IGF1R* mRNA was found to be up-regulated in tumour tissue and stimulated CRC monocytes from early stages revealing a role of *IGF1R* in tumour initiation [41]. Importantly, we have shown that the expression of *IGF1R* mRNA is up-regulated in WBC from the carriers and down-regulated in WBC from the breast cancer patients (p = 0.025). This gives further evidence for the potential predisposition of the carriers for developing breast cancer.

To shed more light on this possible predisposition to breast cancer, we investigated the protein plasma signature in the carriers group and compare it to that in the breast cancer cases and controls. We have identified 35 differentially expressed proteins in plasma from the carriers, breast cancer patients and controls. 10 out of those proteins showed similar expression pattern between the carriers and the patients. The expression of one of these proteins, Apolipoprotein CIII, was found to be reduced in the plasma from pancreatic cancer patients as compared to controls making it a potential marker for the early detection of this disease [42, 43]. Intriguingly, we have shown here that the expression of Apolipoprotein CIII is down regulated more than 3 fold in plasma from breast cancer patients and about 1.5 fold in plasma from the carriers as compared to its level in the controls plasma. This provides another evidence for the potential predisposition of the carriers for developing breast cancer.

## Conclusions

We have shown her that healthy women with *BRCA1* methylation in their WBC have, besides *BRCA1* methylation, modulation in the methylation and expression of other breast cancer-related genes, in addition to the secretion of important cancer- related proteins. We propose the presence of a strong correlation between aberrant methylation of *BRCA1* promoter in WBC, regardless of its presence or absence in breast tissue, and breast cancer-related molecular changes, which may advocates the risk for developing breast cancer. Our findings, together with previous study [19], strongly suggest the potential use of *BRCA1* methylation as a powerful biomarker for detecting predisposed women at a far early age.
